# The chemotherapeutic agent bortezomib induces the formation of stress granules

**DOI:** 10.1186/1475-2867-10-12

**Published:** 2010-04-29

**Authors:** Marie-Josée Fournier, Cristina Gareau, Rachid Mazroui

**Affiliations:** 1 Centre de recherche de l'hôpital St-François d'Assise (CHUQ/CRSFA), 10 rue de l'Espinay, Quebec, QC G1L 3L5, Canada; 2 Département de biologie médicale, Université Laval, Quebec, Canada

## Abstract

**Background:**

Cytoplasmic stress granules (SGs) are specialized storage sites of untranslated mRNAs whose formation occurs under different stress conditions and is often associated with cell survival. SGs-inducing stresses include radiations, hypoxia, viral infections, and chemical inhibitors of specific translation initiation factors. The FDA-approved drug bortezomib (Velcade^®^) is a peptide boronate inhibitor of the 26S proteasome that is very efficient for the treatment of myelomas and other hematological tumors. Solid tumors are largely refractory to bortezomib. In the present study, we investigated the formation of SGs following bortezomib treatment.

**Results:**

We show that bortezomib efficiently induces the formation of SGs in cancer cells. This process involves the phosphorylation of translation initiation factor eIF2α by heme-regulated inhibitor kinase (HRI). Depletion of HRI prevents bortezomib-induced formation of SGs and promotes apoptosis.

**Conclusions:**

This is the first study describing the formation of SGs by a chemotherapeutic compound. We speculate that the activation of HRI and the formation of SGs might constitute a mechanism by which cancer cells resist bortezomib-mediated apoptosis.

## Background

The proteasome is a large multi-subunit complex responsible for the degradation of various proteins, including cell cycle regulators and apoptotic factors, by ubiquitin-dependent and -independent mechanisms [1,2]. Proteasome inhibitors are known to induce apoptosis in proliferating cells [3-6]. The proteasome inhibitor bortezomib is now FDA-approved and in clinical use against mantle cell myeloma and multiple myeloma, against which it displays strong anti-tumor activity [7-14]. However, solid tumors of various histological origins are refractory to bortezomib treatment, and this resistance is also observed in cancer cell lines derived from solid tumors *in vitro *[7,15-17]. The mechanisms by which cancer cells resist bortezomib are still largely unknown, although that this resistance is thought to involve the activation of a general stress response [7,15-17].

When exposed to environmental stress, cells rapidly activate pathways generating a coordinated response involving mRNA translation and turnover, that confers protection against stress-induced damage and promotes their survival. Noxious conditions (e.g. heat shock, oxidative stress, UV radiations, viral infections, etc.) induce cellular arrest of translation initiation [18]. This translational block is largely due to phosphorylation of translation initiation factor eIF2α [19]. Under normal growth conditions, eIF2 associates with initiator Met-tRNA_i_^Met ^(aminoacylated initiator methionyl-tRNA) and GTP, and participates in the ribosomal selection of the start codon. As a prelude to the joining of the small and large ribosomal subunits, GTP complexed with eIF2 is hydrolysed to GDP, and eIF2-GDP is released from the translational machinery. The GDP-bound eIF2 is recycled to the active eIF2-GTP by a reaction catalyzed by the guanine nucleotide-exchange factor, eIF2B. Stress-induced phosphorylation of eIF2a at Ser51 changes this translation factor from a substrate to an inhibitor of eIF2B. Since intracellular levels of eIF2B are approximately 10-20% of those of eIF2 in the cytoplasm, phosphorylation of as little as 10% of eIF2a can be sufficient to sequester virtually all the available eIF2B, thereby blocking the nucleotide exchange activity of eIF2B and therefore inhibiting protein synthesis [20,21]. In metazoans, eIF2a is known to be specifically phosphorylated at Ser51 by at least four kinases that monitor stress response [18], namely PKR, which is mainly activated by viral infection; protein kinase receptor-like endoplasmic reticulum kinase (PERK), which is activated during endoplasmic reticulum stress; GCN2, a protein monitoring amino acid levels in cells and responding to amino acid deprivation and proteasome inhibition; and heme-regulated inhibitor kinase (HRI), which senses osmotic stress, heat shock and oxidative stress produced by arsenite. Stress-induced phosphorylation of eIF2α inhibits translation initiation by stalling translation initiation complexes in an inactive form. The accumulation of such stalled complexes is believed to promote the formation of stress granules [22-24].

Stress granules (SGs) are cytoplasmic ribonucleoprotein-containing bodies whose formation is favored by various stress conditions leading to eIF2α phosphorylation. These include UV irradiation [25], hypoxia [26], arsenite [27-29], and viral infections [30,31]. Since these stress agents are known to inhibit translation initiation, it has been speculated that SGs might represent sites where translation of specific mRNAs is repressed [24]. SGs could repress translation in part by disrupting the interaction of mRNAs with translating ribosomes. A potential role of SGs in translation repression is supported by the observation that specific mRNAs are inefficiently repressed when RNA-binding proteins that contribute to SGs formation are altered [25,28,32-34]. SGs also contain small ribosomal subunits, translation initiation factors and signaling molecules [23,35]. Consistent with the proposed role of SGs as temporary storage or triage sites for untranslated mRNAs, large ribosomal subunits are absent from these foci [27]. Once the inducing stress is relieved, SGs gradually disassemble, which allows translation to resume, a condition essential for cell survival. It is thus postulated that the formation of SGs is central to the stress response by contributing to the reprogramming of gene expression which is essential for cell survival [23]. It is however only during the last few years that the pathological importance of SGs formation in cancer cell resistance to apoptosis became apparent. Indeed, the induction of SGs upon exposure to hypoxia [36], or oxidative stress (e.g. arsenite) [37] leads to tumor cell resistance to apoptosis. One underlying mechanism appears to involve the sequestration and inactivation of pro-apoptotic factors in SGs. The formation of SGs induced by hypoxia in cancer cells has been shown to inhibit apoptosis mediated by the anticancer drug etoposide. This effect was attributed to the sequestration of the signaling scaffold protein RACK1 into SGs, thus leading to the suppression of stress-responsive MAP kinase apoptotic pathways [36]. Other mechanisms by which SGs antagonize apoptosis could involve their sequestration of mRNAs encoding key anti-apoptotic factors, thus preventing degradation of the latter [25]. Although SGs have been shown to form following some types of radiotherapy, whether the formation of SGs can be triggered by chemotherapeutic agents such as bortezomib has not been investigated.

We have previously shown that the formation of SGs in response to the proteasome inhibitor MG132 involves phosphorylation of eIF2α at Ser51 [33]. We found that mouse embryonic fibroblast (MEFs) eIF2α^S51A^, in which eIF2α Ser51 has been mutated to Ala, fail to form SGs upon MG132 treatment. We had also implicated the GCN2 kinase phosphorylating eIF2α in the formation of those SGs. We reported that GCN2-/- MEFs cells have decreased phosphorylation of eIF2α and fail to assemble SGs following MG132 treatment. In the study herein, we now show that bortezomib efficiently induces the formation of SGs in cancer cells. This response involves the phosphorylation of eIF2α by HRI, as shown by the inhibition of bortezomib-induced SGs upon HRI depletion, which also promotes apoptosis. This is the first study describing the induction of SGs by a chemotherapeutic compound. We speculate that the activation of HRI and the resulting formation of SGs might constitute a mechanism by which cancer cells resist bortezomib-mediated apoptosis.

## Results

### Bortezomib induces the formation of SGs in cancer cells

**Figure 1:**
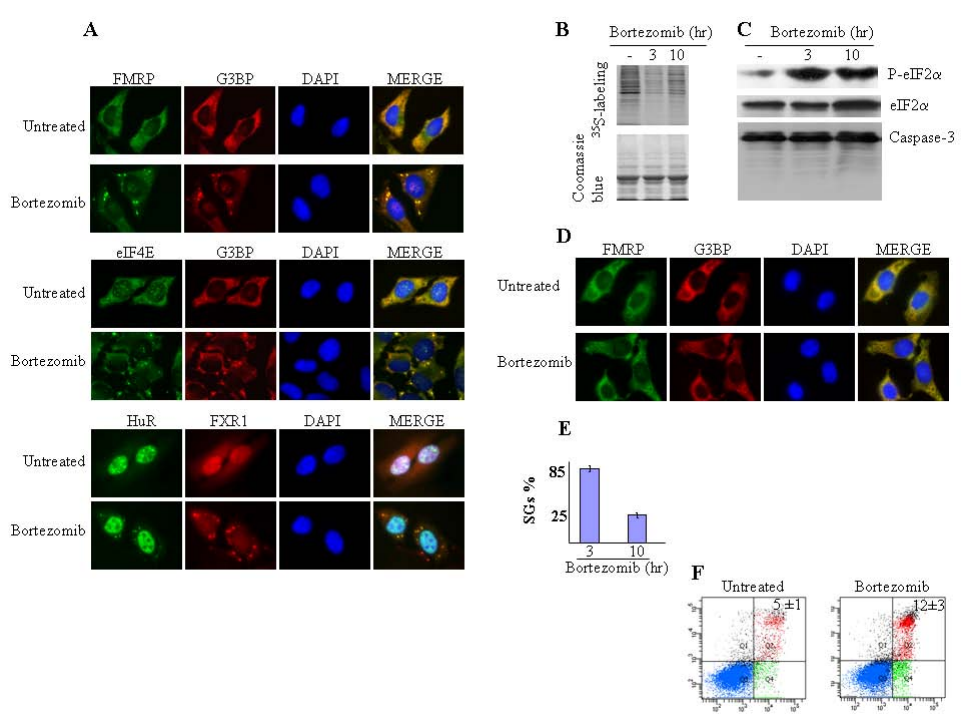
**Bortezomib induces SGs formation**. (A) HeLa cells were treated with 1 μM bortezomib for 3 h, fixed, permeabilized, and processed for immunofluorescence using antibodies against different SG markers. DAPI is used as a marker for nuclei. Pictures were taken using a 100× objective. (B) HeLa cells were treated with 1 μM bortezomib for the indicated times, then incubated with [^35^S] methionine (50 μCi/ml) for another 30 min. Proteins were resolved on a SDS-polyacrylamide gel, stained with Coomassie Blue (*bottom *panel), and detected by autoradiography (*top *panel). (C) HeLa cells were treated with 1 μM bortezomib for the indicated times, and the level of phospho-eIF2α was analyzed by Western blotting using antibodies specific to the phosphorylated form (*top *panel). Detection of total eIF2α levels is shown in the *middle *panel and serves as a loading control. The activation of caspase-3 was analyzed using anti-caspase-3 antibodies (*bottom *panel). (D) HeLa cells were treated with 1 μM bortezomib for 10 h, fixed, permeabilized, and processed for immunofluorescence using antibodies against different SG markers. (E) The indicated histograms represent the percentage of cells harboring SGs (≥5 granules per cell) and is representative of the analysis of five different fields in three independent experiments for a total of 1000 cells. (F) Untreated HeLa cells or cells treated with bortezomib for 24 h were collected, stained with annexin V-FITC and PI, and analyzed by flow cytometry. The percentage of total dead or dying cells (indicated at the top of each panel) was defined as the sum of early (lower right box) and late (upper right box) apoptosis and is presented as the mean ± SEM from 2 independent experiments.

We first assessed whether proteasome inhibition by bortezomib could induce the formation of SGs. Indeed, bortezomib (1 μM, 3 h) efficiently induced SGs in HeLa cells, as assessed by immunofluorescence using various SG markers, namely fragile × mental retardation protein (FMRP), RasGAP-associated endoribonuclease (G3BP), the RNA-binding protein HuR, eIF4E, and fragile × mental retardation syndrome-related protein 1 (FXR1) (Fig. 1A and 1E). Similar results were obtained using other cancer cells such as Calu-I (lung cancer) and Caco (colon cancer) cells (see below and data not shown). In contrast, Hs578T breast cancer cells failed to form SGs in response to bortezomib (see below). Thus, the formation of SGs upon bortezomib treatment is not restricted to HeLa cells and does not occur in all cancer cells. As expected, the formation of SGs in HeLa cells correlated with a reduction of global translation as measured by metabolic labeling (Fig. 1B) and an induction of eIF2α phosphorylation (Fig. 1C, top panel). Bortezomib-induced SGs are reversible since they disassemble following prolonged treatment with bortezomib (1 μM, 10 h) (Fig. 1D-E), allowing a partial recovery of global translation (Fig. 1B). Intriguingly, the disassembly of SGs and the associated translation recovery do not require dephosphorylation of eIF2α since phosphorylation of this factor remained high after prolonged bortezomib addition (Fig. 1C, *top *panel). Moreover, this disassembly of SGs was not a consequence of apoptosis, as assessed by the lack of activation of caspase-3, the main effector of caspase-dependent apoptosis (Fig. 1C, *bottom *panel), and negative results in the annexin V staining assay (Fig. 1F). The latter results indicate that HeLa cells are resistant to bortezomib-mediated apoptosis. Overall, our results show that bortezomib induces the formation of SGs.

### Depletion of HRI prevents bortezomib-induced SGs

**Figure 2:**
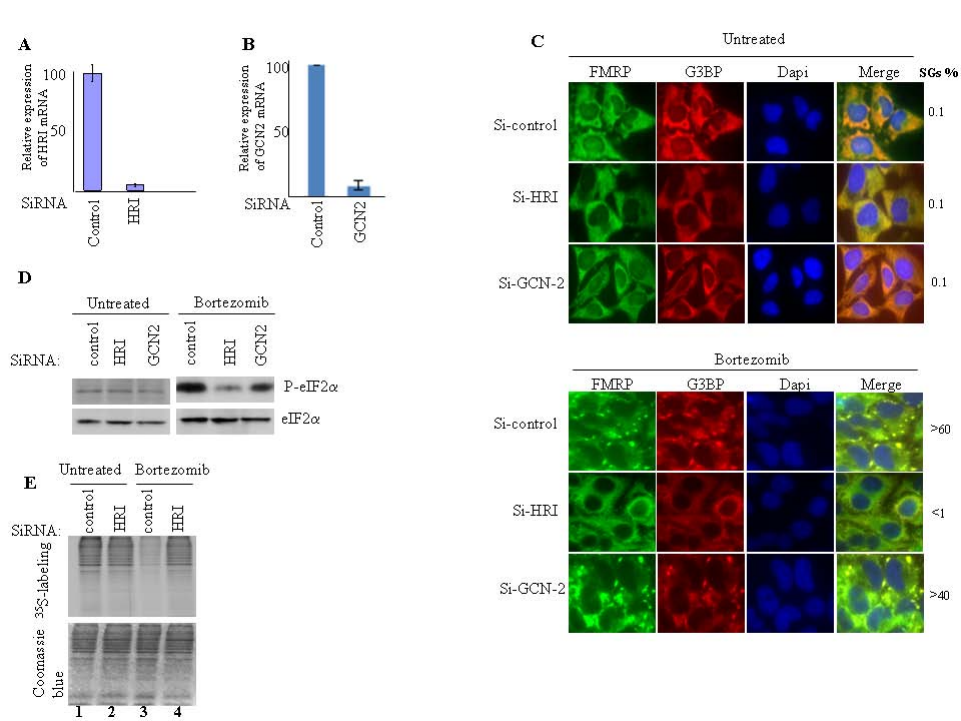
**Reducing HRI levels by siRNA prevents bortezomib-induced SGs formation**. (A, C) HeLa cells were transfected for 48 h with anti-HRI (HRI-1) or anti-GCN2 siRNAs (GCN2-1), or with a control siRNA. (A, B) q(RT)-PCR of HRI (A) and GCN-2 mRNAs (B). Transfected cells were collected and their mRNA content was isolated. The amount of HRI and GCN-2 mRNAs relative to GAPDH mRNA was quantified by real-time q(RT)-PCR using the ΔΔCt method. The results are presented as the mean of triplicate measurements, with error bars corresponding to the SEM. (C) Transfected cells were processed for immunofluorescence using antibodies against different SG markers, as above. (D) HeLa cells were transfected for 48 h with HRI-1, GCN2-1, or with a control siRNA, and then treated with bortezomib for 4 h. Cells were collected and protein extracts were analyzed by Western blot analysis for the amount of phospho- and total eIF2α as described in Figure 1. (E) HeLa cells were transfected for 48 h with HRI-1 siRNA or with control siRNA, and then treated with bortezomib for 3 h before a 30-min incubation with [^35^S] methionine (50 μCi/ml). Proteins were resolved on SDS-polyacrylamide gels, stained with Coomassie Blue (*bottom *panel), and detected by autoradiography (*top *panel).

We have previously implicated the GCN2 kinase as being responsible for eIF2α phosphorylation in the formation of SGs following MG132 treatment [33]. More recently, it was shown that HRI-/- MEFs cells exhibit altered phosphorylation of eIF2α in response to both MG132 and bortezomib [38]. Whether HRI, GCN2, or both are responsible for bortezomib-mediated eIF2α phosphorylation, thus leading to the formation of SGs has not been investigated in cancer cells. To address this question, we first assessed the formation of SGs upon depletion of HRI and GCN2. HeLa cells were treated with specific siRNAs directed against HRI (HRI-1), GCN2 (GCN2-1), or with a control siRNA. Due to the absence of suitable anti-HRI antibodies, we assessed the ability of HRI-1 to knock down its target mRNA using real-time quantitative reverse transcription (qRT)-PCR analysis. The results show that HRI-1 efficiently targeted HRI mRNA to degradation (Fig. 2A). GCN2-1 also efficiently depleted GCN2 mRNA as evidenced by (qRT)-PCR (Fig. 2B). The induction of SGs by bortezomid was then assessed by immunofluorescence using specific SGs markers (Fig. 2C). Less than 1% of cells treated with HRI siRNA displayed SGs in response to bortezomib. In contrast, more than 40% of GCN2-1-treated cells formed SGs upon bortezomib treatment. Control siRNA had a marginal effect on SGs formation since > 60% of the cells thus treated formed SGs following bortezomib treatment. These results indicate that HRI depletion prevents the induction of SGs by bortezomib. This effect of HRI depletion is likely due to altered eIF2α phosphorylation as it significantly reduced the extent of eIF2α phosphorylation induced by bortezomib (Fig. 2D). However, the phosphorylation of eIF2α was not completely abolished in HRI-depleted cells following bortezomib treatment. This indicates that other kinases might contribute to the phosphorylation of eIF2α induced by bortezomib, as shown by the slight reduction of that endpoint upon depletion of the GCN2 kinase (Fig. 2D). Our results indicate that under our conditions HRI is the major kinase involved in the phosphorylation of eIF2α induced by bortezomib, with GCN2 also contributing to this modification. This minimal contribution of GCN2 could explain the residual eIF2α phosphorylation observed in HRI-depleted cells. Residual phosphorylation of eIF2α in HRI-/- is however insufficient to trigger either the formation of SGs (Fig. 2C) or the inhibition of general translation (Fig. 2E). This suggests that a threshold in the extent of eIF2α phosphorylation might be required to induce SGs upon treatment with bortezomib. Thus, the phosphorylation of eIF2α seems to be involved in the formation of SGs which is induced by bortezomib. Our data clearly show that this process requires the activity of HRI.

### HRI depletion promotes apoptosis following bortezomib treatment

**Figure 3:**
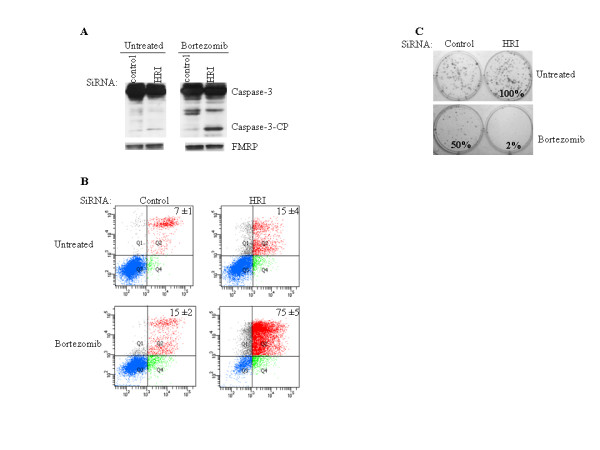
**Reducing HRI levels by siRNA promotes bortezomib-mediated apoptosis**. (A) HeLa cells were transfected for 48 h with HRI-1 or control siRNA, treated with bortezomib for 24 h, and total extracted proteins were analyzed by Western blot using anti-caspase-3 antibodies (*top *panel). CP: cleaved product. FMRP serves as a loading control (*bottom *panel). (B) Following treatment with HRI-1 or control siRNA, HeLa cells were incubated with bortezomib for 24 h, then stained with annexin V-FITC and PI, and analyzed by flow cytometry. The percentage of total dead or dying cells (indicated at the top of each panel) was defined as the sum of early (lower right box) and late (upper right box) apoptosis and is presented as the mean ± SEM from two independent experiments. (C) Following treatment with HRI-1 or control siRNA, HeLa cells were incubated with bortezomib for 24 h, trypsinized, counted, replated in the absence of drug, and incubated for 10 d. Before colony counting, cells were fixed and dried. Populations > 50 cells were counted as one surviving colony. Data were calculated as the percentage of surviving colonies relative to control (untreated) plates. The results are expressed as the mean of triplicate measurements.

Previous studies have reported that MEFs lacking HRI fail to form SGs following treatment with arsenite [39]. Quite significantly, HRI deficiency resulted in cell death during the recovery phase from arsenite treatment. Whether HRI can promote resistance of cancer cells to chemotherapeutic agents has not been investigated. We next addressed the hypothesis that HRI depletion might sensitize HeLa cells to bortezomib-mediated apoptosis. To that purpose, HeLa cells treated with either HRI-1 or control siRNAs were exposed to bortezomib for 10 and 24 h, and apoptosis was then assessed by assaying caspase-3 activation and annexin V staining. Depletion of HRI, which was confirmed by (qRT)-PCR, did not induce significant apoptosis *per se *(Fig. 3A-B). However, HRI depletion induced apoptosis in a high percentage of cells upon bortezomib treatment (Fig. 3A, B).

**Figure 4:**
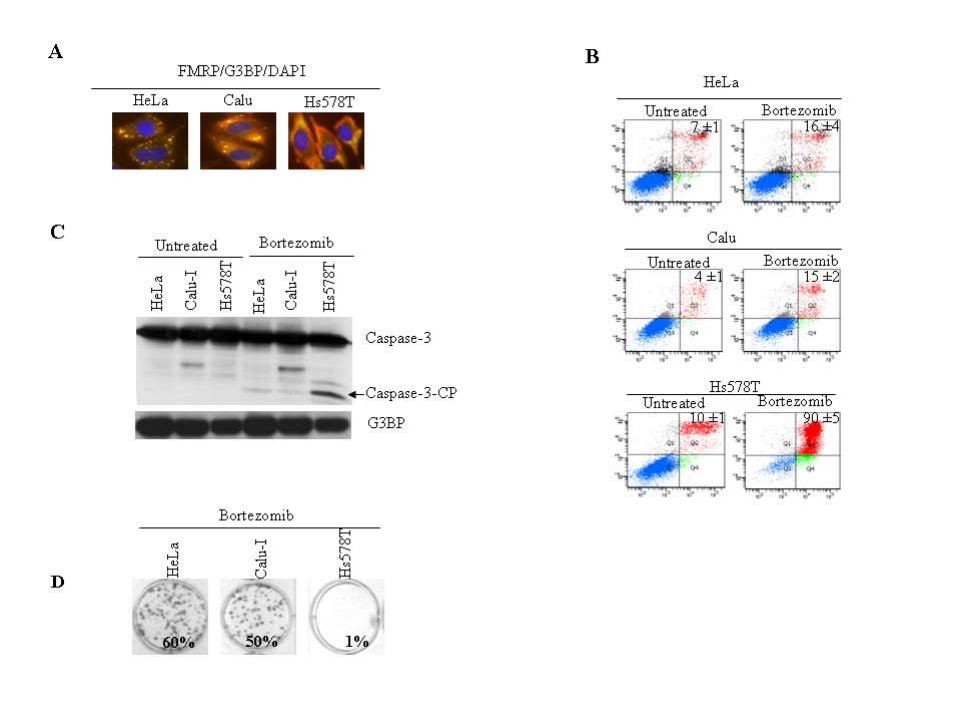
**The formation of SGs correlates with resistance to bortezomib-mediated apoptosis**. HeLa, Calu-1, and Hs578T cells were treated with bortezomib for 3 h (A) or 24 h (B to D). (A) Cells were processed for immunofluorescence to detect SGs using anti-FMRP and anti-G3BP antibodies. (B) Cells were stained with annexin V-FITC and PI, and analyzed by flow cytometry. The percentage of total dead or dying cells (indicated at the top of each panel) was defined as the sum of early (lower right box) and late (upper right box) apoptosis and is presented as the means ± SEM from two independent experiments. (C) Cells were harvested and protein extracts analyzed by Western blot for the activation of caspase-3 using anti-caspase-3 antibodies. G3BP serves as a loading control. (D) Following bortezomib treatment for 24 h, cells were trypsinized, replated in the absence of drug, and incubated for 10 d. Before colony counting, cells were fixed and dried. Populations > 50 cells were counted as one surviving colony. Data were calculated as the percentage of surviving colonies relative to untreated plates. The results are presented as the mean of triplicate measurements.

To further confirm the role of HRI in promoting resistance of cancer cells to bortezomib-induced apoptosis, we performed clonogenic survival assays. For this, HeLa cells were treated with anti-HRI or control siRNAs, incubated with bortezomib and replated in fresh medium for 10 d, at which point colonies were counted. Depleting HRI significantly decreased cell survival and growth following treatment with bortezomib (Fig. 3C). Overall, the results identified HRI as an SGs-promoting factor that confers resistance to bortezomib-induced apoptosis. Since SGs are known to antagonize apoptosis [24], our results suggest that HRI may promote cancer cell resistance to bortezomib, at least in part, by inducing SGs. One corollary of the latter finding is that cells which fail to form SGs following bortezomib treatment might become more susceptible to apoptosis. We assessed this hypothesis using Hs578T cells, which do not form SGs upon bortezomib treatment (Fig. 4A). HeLa and Calu-I cells were used as positive controls. As shown using caspase-3 activation and annexin V staining assays, bortezomib induces a high percentage of apoptosis in Hs578T cells while both HeLa and Calu-I cells are resistant to that treatment (Fig. 4B-C). As expected, bortezomib-mediated apoptosis inhibited Hs578T cell growth after removing the drug, as evidenced by a clonogenic survival assay (Fig. 4D). We obtained similar results using MDA-MB-231 human breast carcinoma cells which do not form SGs upon bortezomib treatment (data not shown). Thus, for the set of cell lines selected here, the formation of SGs in cancer cells correlates with their resistance to bortezomib.

## Discussion

The present study shows for the first time that bortezomib can induce the reversible formation of SGs in cancer cells. The formation of SGs is associated with a reduction of general mRNA translation, while their disassembly following extended incubation with bortezomib correlates with a partial recovery of general translation. SGs production also correlates with the phosphorylation of eIF2α by HRI. Depletion of HRI blocks both the formation of SGs and the chemoresistance of cancer cells to bortezomib. Our studies unveil a specific survival pathway that involves HRI and the formation of SGs, which might be targeted to prevent cancer cell resistance to bortezomib-mediated apoptosis.

It is well known that under different stress conditions, eIF2α phosphorylation triggers SGs formation [23]. Our present work demonstrates that depletion of HRI prevents the formation of SGs and decreases eIF2α phosphorylation induced upon bortezomib treatment. However, although HRI depletion completely prevents the formation of SGs induced by bortezomib, it leaves a residual but significant amount of eIF2α phosphorylation unaffected in that model. That residual level of eIF2α phosphorylation could be due either to an incomplete suppression of HRI protein synthesis, or to the activation of the GCN2 kinase, although we cannot exclude the possible contribution of the other eIF2α kinases [23]. Notwithstanding these observations, the residual phosphorylation of eIF2α observed in HRI-depleted cells treated with bortezomib is clearly insufficient for triggering the formation of SGs. One possibility is that initiating the formation of SGs requires a minimum threshold of eIF2α phosphorylation. Another possibility is that, in the bortezomib model, HRI might promote the formation of SGs via mechanisms other than or in addition to eIF2α phosphorylation. We and others have described the inactivation of the translation initiation factor eIF4A as an alternative pathway for the induction of SGs which occurs independently of eIF2α phosphorylation and in absence of any additional stress [30,40]. We subsequently found that inactivation of other translation initiation factors also induces the formation of SGs in absence of stress [41]. In this context, it is tempting to speculate that HRI may affect other, as yet unknown translation initiation factors in addition to phosphorylating eIF2α, thus triggering the formation of SGs upon bortezomib treatment. Other pathways whose inactivation impairs the induction of SGs by stress agents also include microtubule polymerization and the acetylglucosamination (O-GlcNAc) modification of ribosomal proteins [24]. It will be interesting to investigate the role of these pathways in the formation of SGs and test if HRI depletion could affect those pathways in cells treated with bortezomib.

Previous studies have shown that HRI-deficient MEFs fail to form SGs upon arsenite treatment [39]. This effect of HRI knockout is physiologically relevant since it resulted in massive cell death during the recovery phase from arsenite treatment [39]. However, this model was not investigated in cancer cells, and more importantly, the role of HRI activation in chemoresistance to bortezomib had never been addressed. Our work has thus unveiled a critical role of HRI in promoting cancer cell resistance to bortezomib, at least in part via the induction of SGs. HRI is known to be highly expressed in erythroid cells, although minimal expression of HRI is also found in a wide range of non-erythroid cells [42,43]. It is thus intriguing that HRI plays a critical role in promoting resistance of non-erythroid cells such as HeLa to apoptosis. One possibility is that the expression and/or activation of HRI might be altered in cancer cells of non-erythroid origin. Our q(RT)-PCR analysis indicates that HRI mRNA is indeed abundant in HeLa cells and its high expression remains unaltered by bortezomib treatment (data not shown). It will be interesting to compare the expression of HRI in bortezomib-resistant and -sensitive cancer cells. The mechanism of HRI activation by bortezomib is currently unknown but may involve an interaction with heat shock proteins. Hsp70 and Hsp90 are two heat shock proteins which act as molecular chaperones to modify the conformation of other proteins [44]. In reticulocytes, Hsp70 and Hsp90 were shown to activate HRI following arsenite treatment [45]. In addition to their role of chaperones, overexpression of Hsp70 and Hsp90 in cancer cells confers protection against apoptosis. However, depletion of the inducible form of Hsp70 (Hsp72) does not impair the induction of SGs by bortezomib (data not shown), indicating that this protein may not be involved in the activation of HRI under our conditions. The role of Hsp90 in promoting bortezomib-induced HRI activation, and the formation of SGs remains to be investigated.

How could the formation of SGs promote cancer cell resistance to bortezomib-mediated apoptosis? SGs could enhance this survival pathway by sequestering untranslated mRNAs to free the ribosomes needed for the efficient and rapid translation of mRNAs encoding anti-apoptotic factors [22]. In addition, SGs might promote tumor cell survival by preventing the degradation of mRNAs encoding key survival proteins, as reported by Moeller *et al. *[25]. In the latter study, it was shown that radiotherapy induces the formation of SGs where mRNAs encoding anti-apoptotic cytokines such as VEGF are recruited and accumulated. Following reoxygenation, however, SGs disassemble, thus releasing those mRNAs that are then translated in large amounts. The ensuing overproduction of cytokines then causes radioresistance of the tumor and its subsequent regrowth. This mechanism could also explain the effects of depleting two SGs-promoting factors, TIA and HDAC6, in preventing cell survival. In these studies, depletion of either TIA or HDAC6 was shown to prevent arsenite-induced SGs formation, thus promoting apoptosis during recovery from arsenite treatment [37,39]. Other mechanisms by which SGs prevent cancer cell resistance to bortezomib might involve the sequestration and inactivation of key apoptotic signaling molecules such as RACK1 or TRAF2 [36,46], thus preventing the initiation of apoptotic cascades. Clearly, further studies are needed to determine if the formation of SGs is a key pathway leading to chemoresistance to bortezomib.

## Conclusions

The present study has established HRI as a critical factor promoting chemoresistance *in vitro*. Consideration should be taken about developing compounds to target HRI, which could be combined with bortezomib to treat chemoresistant cancers. We also provide a framework for further documenting the therapeutic relevance of targeting the formation of SGs as a new tool to chemosensitize cancer cells.

## Methods

### Cell lines and culture

HeLa, Calu-1 (lung cancer), and Hs 578T (breast cancer) cells were obtained from the American Type Culture Collection (Manassas, VA). Cells were cultured in DMEM supplemented with 10% fetal bovine serum, penicillin, and streptomycin. All supplements for cell culture were from Sigma-Aldrich (St. Louis, MO).

### Drugs and drug treatments

Bortezomib was purchased from LC Laboratories and dissolved in DMSO to a 65 mM stock solution, and stored at -20°C. Bortezomib treatment was performed when cells had reached 60-80% confluence.

### [^35^S]Methionine labeling

Cells in 6-well plates were labeled for 30 min with 1 ml methionine-free DMEM (Sigma) supplemented with 10% fetal bovine serum and 50 μCi/ml of [^35^S] methionine (Easy Tag, PerkinElmer/NEN Radiochemicals).

### Antibodies

Anti-caspase-3, phospho-specific anti-eIF2α and the pan anti-eIF2 antibodies were purchased from Cell Signaling Technology (Beverly, MA). Anti-HuR, anti-G3BP, anti-FMRP, anti-FXR1, and anti-eIF4E have been previously described [30,33,41].

### siRNA transfections

All siRNAs were purchased from Dharmacon. siRNA transfections were performed in HeLa cells essentially as previously documented [47] using Hiperfect reagent according to the manufacturer's protocol (Qiagen and Dharmacon). Twenty-four hours before transfections, cells were trypsinized and plated to obtain 60-80% confluence the day after. For a 6-well plate, annealed duplexes were used at a final concentration of 50 nM. Forty eight hours post-transfection, cells were either fixed and processed for immunofluorescence or harvested for protein and mRNA extraction.

### Fluorescence microscopy

Immunofluorescence experiments were performed as previously described [29]. Briefly, following fixation and permeabilization, cells were incubated with primary antibodies diluted in 0.1% Tween-20 in phosphate-buffered saline (PBS) for 1 h at room temperature. After washing, cells were incubated with goat anti-mouse/rabbit IgG (H+L) secondary antibodies coupled to Alexa Fluor 488/594. Fluorescence was visualized using an Olympus fluorescence microscope equipped with AxioCam HR digital camera and the the Axiovision acquisition software. Images were compiled using Adobe Photoshop (Adobe Systems, Mountain View, CA).

### Real-time quantitative RT-PCR

RT-PCR reactions were performed using the Quantitect Reverse Transcription kit (Qiagen). Each reaction was performed by mixing 2 μl of RNA at 500 ng/μl, 10 μl of RNase-free water, 2 μl of gDNA Wipeout Buffer 7×, 4 μl of Quantiscript RT Buffer 5×, 1 μl of RT Primer Mix and 1 μl of Quantiscript Reverse Transcriptase.

Real-time PCR reactions were carried out using the Power SYBR Green PCR Master mix (Applied Biosystems) in a total volume of 25 μl: 12.5 μl of PCR Master Mix, 0.67 μl of forward primer at 3.75 μM, 0.67 μl of reverse primer at 3.75 μM, 9.2 μl of deionized water and 2 μl of RT-PCR. Reactions were run and data analyzed on the MX3000 QRT PCR system (Applied Biosystems) with a 4-stage program: first stage: 2-min incubation at 50°C; second stage: 10-min incubation at 95°C, followed by a 2-step reaction in the third stage: 95°C × 15 s and 55°C × 60 s for 40 cycles; and a fourth stage made of a 3-step reaction (95°C × 15 s, 60°C × 20 s and 95°C × 15 s).

For preparing templates for the HRI mRNA, the oligonucleotide pairs used were: 5'-GCCCTGATCAGCCAAGTAAAA-3' (forward primer), and 5'-TCTGGACGAGTATGTGTTGGTG-3' (reverse primer). For preparing templates for the GCN2 mRNA, the oligonucleotide pairs used were: 5'-CAAGGCCTAACTGGTGAAGA-3' (forward primer), and 5'-AGGTAGGTGGGCATTTAACC-3' (reverse primer). For preparing templates for the glyceraldehyde 3-phosphate dehydrogenase (GAPDH) mRNA, the oligonucleotide pairs used were: 5'-ACGACCACTTTGTCAAGCTC-3' (forward primer), and 5'-GTTGCTGTAGCCAAATTCGT-3' (reverse primer).

### Annexin V-fluorescein isothiocyanate/propidium iodide assay

Following treatments, both adherent and detached cells were harvested. Cells were washed with ice-cold PBS, then pelleted again at 1500 rpm for 10 min at 4°C, and resuspended in ice-cold binding buffer (10 mM HEPES/NaOH, pH 7.4, 140 mM NaCl, 2.5 mM CaCl2). The cells were subsequently stained with annexin V-fluorescein isothiocyanate (FITC) and propidium iodide (PI) for 15 min in the dark. A total of 50,000 cells were counted, and dead cells were analyzed by flow cytometry.

### Clonogenic survival assay and annexin V analysis

Cells were plated in duplicate and incubated for 24 h. Following treatments, cells were washed with PBS, trypsinized, counted, replated (1 × 103 cells per well in 6-well plates) in the absence of drug, and incubated for 10 d. Before colony counting, cells were washed with PBS, stained with 0.1% (w/v) crystal violet in PBS containing 0.0037% (v/v) formaldehyde, rinsed with deionized water and dried. Populations > 50 cells were counted as one surviving colony.

## Competing interests

The authors declare that they have no competing interests.

## Authors' contributions

All authors read and approved the final manuscript. MJF and CG have equally contributed to this work.
